# Characteristics and prevalence of anxiety and depression symptoms among patients diagnosed with obstructive sleep apnea in a tertiary care center

**DOI:** 10.5339/qmj.2024.qitc.21

**Published:** 2024-04-25

**Authors:** Asma Albtoosh, Mohammed Aloqaily, Moayad Shaf’ei, Shahed Alqudah, Omar Ihmoud, Mohammad Sharayah, Ensherah Mokheemer, Omar Ifdielat, Dunia Z. Jaber, Wafi Aloqaily, Amro Alradaideh

**Affiliations:** 1 Faculty of Medicine, University of Jordan, Amman, Jordan Email: asmaalbtoosh@gmail.com; 2 Internal Medicine Department, Hamad Medical Corporation, Doha, Qatar

**Keywords:** Obstructive Sleep Apnea, Psychiatry, Apnea Hypopnea Index, Depression, Anxiety, Obesity

## Background

Obstructive sleep apnea (OSA) is a syndrome marked by recurrent upper airway obstructions causing intermittent hypoxia and sleep arousal. It is a debilitating multi-systemic condition associated with various diseases including psychiatric disorders such as depression and anxiety.^1^

## Methodology

This cross-sectional study was designed to assess patient characteristics and the prevalence of anxiety and depression in individuals diagnosed with OSA at Jordan University Hospital. Following consent, demographic details and OSA symptoms were collected from patients and medical records over 6 months. Additionally, the levels of anxiety and depression were screened using a validated and translated version of the Hospital Anxiety and Depression Scale (HADS).^2^

## Results

**Figure 1.**
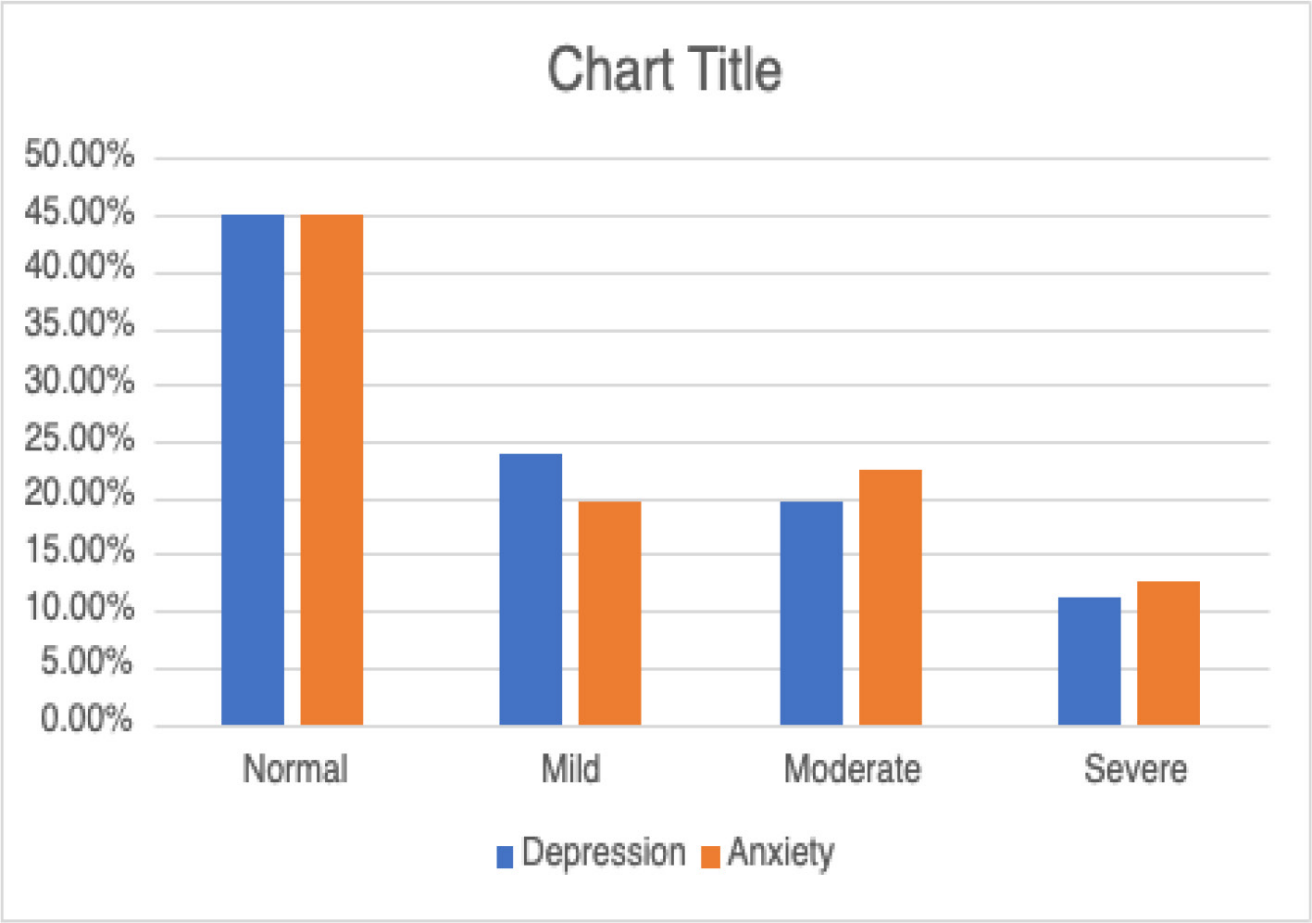
Prevalence of anxiety and depression in OSA patients.

A total of 71 patients were included, with a female-to-male ratio of 1.1:1, a mean age of 50.44 years, and a BMI of 37.53 kg/m^2^. Notably, the average duration from symptom onset to presentation was 2.97 years, with snoring being the predominant symptom at presentation (53.52%), followed by sleep apnea (38.03%) and dyspnea (21.13%). During follow-up, snoring, daytime sleepiness, and dyspnea were the most commonly reported symptoms (91.55%, 73.24%, and 69.01%, respectively), while hypertension emerged as the predominant comorbidity (61.97%). A history of smoking (current/past) was noted in 35.2% and a family history of OSA was observed in only 14.08%. Importantly, the majority of patients did not have anxiety or depression (45.07%) based on the HADS score. However, 22.53% had moderate anxiety, followed by severe and mild anxiety in 19.72% and 12.68%, respectively. For depression, mild cases accounted for 23.94%, surpassing moderate (19.72%) and severe (11.27%) cases based on the HADS (Figure 1).

## Conclusion

This study highlights the significance of mental health considerations in OSA patients, revealing a prevalence of anxiety and depression consistent with the existing literature, with no consensus yet.^3^ The forthcoming discussion will focus on comparisons with prior research, impact on clinical practice, and proposed directions for future investigations.

## Conflict of Interest

Authors declare no conflict of interest.
